# The relationship between marital satisfaction and depression in infertile couples: an actor–partner interdependence model approach

**DOI:** 10.1186/s12888-018-1893-6

**Published:** 2018-09-25

**Authors:** Saman Maroufizadeh, Mostafa Hosseini, Abbas Rahimi Foroushani, Reza Omani-Samani, Payam Amini

**Affiliations:** 1 Department of Epidemiology and Biostatistics, School of Public Health, Tehran University of Medical Sciences, Tehran, Iran; 2 Department of Epidemiology and Reproductive Health, Reproductive Epidemiology Research Center, Royan Institute for Reproductive Biomedicine, ACECR, Tehran, Iran

**Keywords:** Depression, Marital satisfaction, Infertility, Actor–partner interdependence model

## Abstract

**Background:**

Much evidence consistent with the Marital Discord Model of Depression (MDMD) suggests that marital discord is associated with depression, but no studies examine the relationship between marital satisfaction and depression at the dyadic level in infertile couples. This study examined the effect of actors’ and partners’ marital satisfaction on depressive symptoms in husband-wife dyads with infertility using an innovative dyadic analysis approach, the Actor–Partner Interdependence Model (APIM).

**Methods:**

In this cross-sectional study, the sample comprised of 141 infertile couples in the evaluation phase of treatment. We collected data in a referral infertility center in Tehran, Iran between February and May 2017. Marital satisfaction and depression were measured using ENRICH marital satisfaction scale and Hospital Anxiety and Depression Scale, respectively, before starting the treatment. Dyadic analysis applying the APIM was used. In this study, actor effect is the impact of a person’s marital satisfaction on his/her own depression. Partner effect is the impact of a person’s marital satisfaction on his/her partner’s depression.

**Results:**

The APIM analysis revealed that both men and women’s marital satisfaction excreted an actor effect on their own depression (β = − 0.412, *P* < 0.001; β = − 0.263, *P* = 0.002, respectively). Furthermore, men’s marital satisfaction exerted a significant partner effect on their wives’ depression symptoms (β = − 0.170, *p* = 0.047). However, the wives’ marital satisfaction was not related to their husbands’ depressive symptoms (β = − 0.028, *P* = 0.735).

**Conclusions:**

The findings support that the MDMD is a valid theoretical model for the conceptualization of marital satisfaction and depressive symptoms among infertile couples and suggest that interventions to reduce depressive symptoms should include both men and women.

## Background

Infertility is medically defined as “the failure to achieve a clinical pregnancy after 12 months or more of regular unprotected sexual intercourse” [1]. It is a global public health concern affecting 9% of reproductive-aged couples worldwide [2] with negative psychological consequences. One of the most often-cited repercussions of infertility is depression disorder. For example, in two studies conducted by Maroufizadeh et al. [3] and Omani-Samani et al. [4] in Iran, the prevalences of depression were 33.0% and 30.5%, respectively, which are higher than what was reported in general population. Some of the demographic/fertility risk factors for depression in infertile couples include educational level, cause of infertility, duration of infertility, and having failure in previous infertility treatments [3–5]. One the other hand, infertility and its treatment has also a negative impact on a person’s marital satisfaction, relationship satisfaction, and sextual functioning [6–8].

Based on the Marital Discord Model of Depression (MDMD) [9], marital discord is an important risk factor for depressive symptoms in married couples. A growing body of evidence support the MDMD. For example, in a meta-analysis of 26 cross-sectional study, the effect size of marital satisfaction on depression was − 0.42 for women and − 0.37 for men [10]. In addition, longitudinal studies have found that marital dissatisfaction is associated with subsequent depressive disorder [11, 12]. On the other hand, marital satisfaction is strongly influenced by sexual functioning. Satisfying marital and sexual functioning protects against the development of psychological distress but is also a factor related to depression and anxiety [13, 14].

A fundamental assumption in behavioral and social science statistical methods is the independence of observations. Many of the phenomena studied in this context are dyadic in nature (e.g., research on man-woman dyads). The observations arising from such designs are not independent, but interdependent (i.e., the characteristics of one member of the dyad affects outcomes of the other member in the dyad) [15, 16]. In this case, conventional statistical methods are not proper for analyzing data; Instead, the methods that take into account the interdependence is required [16]. To address this issue, Kenny et al. [16] developed the Actor Partner Interdependence Model (APIM), the most frequently used analytical model of dyadic data. This model simultaneously estimates the effects of an individual’s attributes on his/her own outcome variable (actor effect), as well as his/her partner’s (partner effect).

Most studies examining the effect of marital satisfaction on depression in infertile couples use the conventional statistical methods. Although valuable, these studies fail to take into account the interdependency of couples’ data, and consequently fail to show interpersonal relationships. In addition, since experience of infertility is a shared problem between members of a couple, examining the interpersonal relationship is especially relevant [17]. Due to these reasons, in the last few years, APIM framework has been used to examine many relationship processes, including the effect of depression on quality of life in Iranian infertile couples [18], and effects of spirituality and infertility-related stress on quality of life in Brazilian infertile couples [19]. The aim of the present study was twofold: (a) to examine whether there were differences in the levels of marital satisfaction and depression between men and women dyads with infertility, and (b) to apply the APIM framework to elucidate and differentiate actor and partner effects of marital satisfaction on depression in infertile couples.

## Methods

### Participants and procedure

This cross-sectional study was performed at the infertility treatment clinic of Royan Institute, Tehran, Iran. This clinic is one of the largest clinics for infertility treatment in Iran [20]. Infertile couples come to this center, not only from the capital of Iran but also from all around the country. The data were collected in the evaluation phase of treatment using the convenience sampling method between February and May 2017. Couples had to meet the following criteria to be eligible for the study: (1) married couple; (12) 18 years or older; (2) willingness to take part in the study; (3) experiencing fertility problems; and (4) ability to read, write, and comprehend Persian. Both husband and wife were asked to complete the measures with no discussion between them. In total, 141 infertile couples agreed to participate and filled out the instruments completely (response rate: 82.9%).

### Measures

#### Marital satisfaction

Marital satisfaction was measured using the 10-Item ENRICH Marital Satisfaction Scale (EMS Scale). The EMS Scale is a 10-item self-report inventory that measures marital satisfaction [21]. Items are rated on a 5-point Likert scale, ranging from 1 (strongly disagree) to 5 (strongly agree). Total scores range from 10 to 50, with higher scores indicating greater marital satisfaction. In the current study, the Cronbach’s alpha coefficients of EMS Scale for men and women were 0.752 and 0.790, respectively.

#### Depression

Depression symptoms was measured using the Hospital Anxiety and Depression Scale (HADS). The HADS is a frequently used 14-item self-report inventory composed of two 7-items subscales: anxiety (HADS-A) and depression (HADS-D) [22]. Items are rated on a 4-point Likert scale, ranging from 0 (no symptoms) to 3 (severe symptoms). Both subscales scores range from 0 to 21, with higher scores indicating greater levels of anxiety and depression. The Persian version of the HADS has shown satisfactory reliability and validity in infertile people [23] and has been used in many studies involving infertile people [3, 24]. In this study, we used only the HADS-D subscale. In the current study, the Cronbach’s alpha coefficients of HADS-D subscale for men and women were 0.708 and 0.722, respectively.

### Statistical analysis

#### Preliminary analyses

Comparison of demographics/fertility information, marital satisfaction and depression for husbands and wives were done using the McNemar test and paired t test. Pearson’s correlation coefficient was used to determine the relationship among the study variables.

#### APIM analysis

**Fig. 1:**
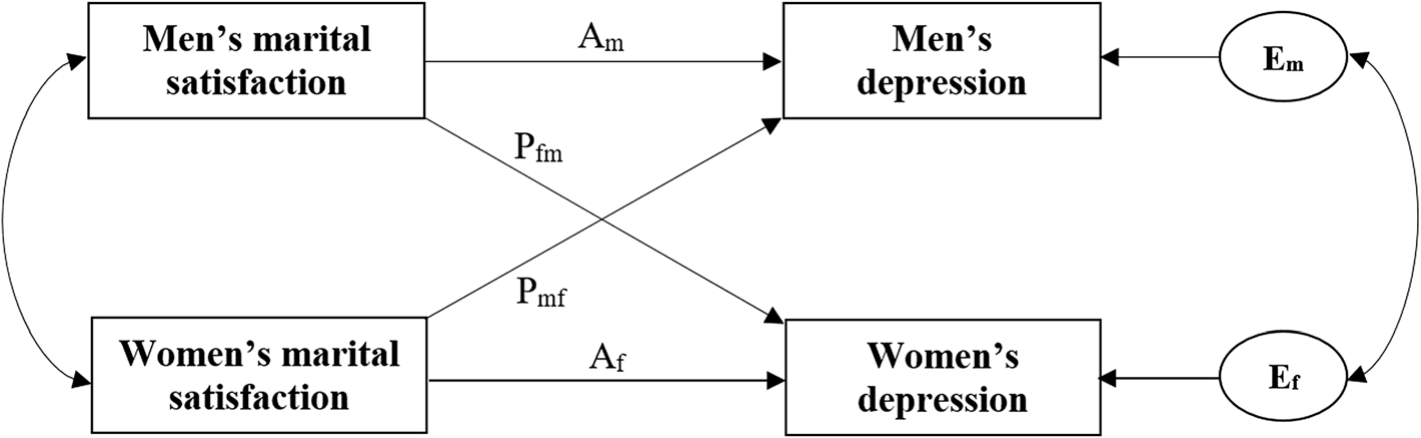
Actor–Partner Interdependence Model of marital satisfaction and depression. Legend 1: A_m_: actor effect of men’s marital satisfaction on his own depression; A_f_: actor effect of women’s marital satisfaction on her own depression; P_fm_: partner effect of men’s marital satisfaction on women’s depression; P_mf_: partner effect of women’s marital satisfaction on men’s depression; E_m_ and E_f_: residual errors on depression for men and women, respectively

In this study, we analyzed our data using the APIM approach. Figure 1 depicts the APIM framework of a husband-wife dyad in which there is two variables from each in the dyad: marital satisfaction and depression. This approach takes into account the interdependence of couples’ data. Also, with APIM approach, both actor effects (e.g. how a person’s level of marital satisfaction affects his or her own depression) as well as partner effects (e.g. how a person’s level of marital satisfaction affects his or her partner’s depression) can be examined simultaneously.

There are three methods to estimate the APIM parameters: the pooled regression modeling, multilevel modeling, and structural equation modeling (SEM). As recommended by Kenny et al. [16], SEM with distinguishable dyads is the simplest data analytic method for estimating the APIM.

The APIM analysis was done with Mplus software version 6.12 (Muthén & Muthén, Los Angeles, CA, USA), and preliminary analyses were done with IBM SPSS Statistics for Windows, Version 22.0 (IBM Crop., Armonk, NY, USA).

## Results

### Characteristics of the infertile couples

Table 1 shows the demographic and fertility characteristics of the infertile couples. The wives, on average, were 5.10 years younger than their husbands (t _(140)_ = 12.88, *P* < 0.001), but had a similar education level as their husbands (χ^2^_(1)_ = 2.56, *P* = 0.109). The mean duration of marriage and infertility were 7.37 ± 4.40 and 4.85 ± 3.76 years, respectively. Infertility was due to a male or female factor in 36.2 and 21.3% of dyads, respectively. In 19.1%, both male and female factors were observed, and 23.4% of couples had unexplained infertility. Of the couples, 27.7% had secondary infertility, 23.4% had history of abortion and 49.6% were treated for infertility for the first time.

**Table 1:** Demographic and fertility characteristics of the infertile couples (*n* = 141 couples)

	Men	Women	Test statistic	P
Age (years)	34.92 ± 6.35	29.82 ± 6.00	t_(140)_ = 12.88	< 0.001
Educational level			χ^2^_(1)_ = 2.56	0.109
Non-academic	96 (68.1)	85 (60.3)		
Academic	45 (31.9)	56 (39.7)		
Duration of marriage (years)	7.37 ± 4.40	–		
Duration of infertility (years)	4.85 ± 3.76	–		
Cause of infertility				
Male factor	51 (36.2)	–		
Female factor	30 (21.3)	–		
Both	27 (19.1)	–		
Unexplained	33 (23.4)	–		
Failure of previous treatment				
No (First treatment)	71 (50.4)	–		
Yes	70 (49.6)	–		
History of abortion				
No	108 (76.6)	–		
Yes	33 (23.4)	–		
Type of infertility				
Primary	102 (72.3)	–		
Secondary	39 (27.7)	–		

Values are given as “Mean ± SD” or “n (%)”

### Preliminary analyses

Means, SDs, and correlations for study variables are presented in Table 2. According to the paired t test, there were no significant differences between men and women on the depression (t_(140)_ = 0.45, *P* = 0.653) and marital satisfaction (t_(140)_ = 0.09, *P* = 0.925).

**Table 2:** Means, standard deviations, and correlations among study variables (*n* = 141 couples)

	Mean (SD)	1	2	3	4
1 Men marital satisfaction	39.31 (6.56)	1			
2 Men depression	4.94 (3.41)	−0.424^***^	1		
3 Women marital satisfaction	39.26 (6.70)	0.423^***^	−0.203^*^	1	
4 Women depression	5.09 (3.33)	−0.281^***^	0.256^**^	−0.335^***^	1

*SD* Standard Deviation

^*^P < 0.05; ^**^P < 0.01; ^***^P < 0.001

Within-dyad correlations revealed that wives’ and husbands’ scale scores were significantly correlated for depression (*r* = 0.256, *P* = 0.002) and marital satisfaction (*r* = 0.423, *P* < 0.001). Based on Pearson correlations, husbands’ marital satisfaction was correlated with both their own depression (*r* = − 0.424, P < 0.001) and their wives’ depression (*r* = − 0.281, P < 0.001). Wives’ marital satisfaction were also correlated with both their own depression (*r* = − 0.335, *P* < 0.001) and their husbands’ depression (*r* = − 0.203, *P* = 0.016).

### APIM analysis

The APIM analysis indicated that the man’s marital satisfaction as well as woman’s marital satisfaction exhibited an actor effect on their own depression (β = − 0.412, *P* < 0.001; β = − 0.263, P = 0.002, respectively) (Table 3). With regard to partner effects, however, only the man’s marital satisfaction had a partner effect on woman’s depression (β = − 0.170, *p* = 0.047). The partner effect of woman’s marital satisfaction on man’s depression was not significant (β = − 0.028, *P* = 0.735).

**Table 3:** Actor and partner effects of marital satisfaction on depression in infertile couples (*n* = 141 couples)

	Men			Women		
	β (SE)	t	P	β (SE)	t	P
Actor’s marital satisfaction	− 0.412 (0.078)	5.28	< 0.001	− 0.263 (0.084)	3.13	0.002
Partner’s marital satisfaction	−0.028 (0.084)	0.34	0.735	−0.170 (0.085)	1.98	0.047

β: Standardized Coefficient; *SE* Standard Error

## Discussion

To the best of our knowledge, this study is the first of its kind to use the APIM approach to examine the intrapersonal and interpersonal influences of marital satisfaction on depression in a sample of infertile couples. Although the most of studies examining the relationship between marital satisfaction and depressive symptoms focused on the intrapersonal mechanisms (actor effects), there are growing calls to examine the interpersonal mechanisms (partner effects). Since infertility is also a shared problem between spouses, both men and women need to be involved and considered as a dyad in the data analysis. According to within-dyad correlations, considerable correlations between wives’ and husbands’ scores were observed. These findings confirm that husbands and wives’ scores were adequately related to be deemed statistically interdependent, and so APIM approach would be more appropriate than conventional statistical analyses.

In keeping with some previous studies [3, 25], we found that there was no significant difference between men and women in the depression level. However, in a two study conducted in Poland [26] and Pakistan [27], women had a higher level of depression than men. In this study, there was also no sex difference in the marital satisfaction, which is in line with Peterson et al. study [28]. In a study performed by Drosdzol and Skrzypulec [26], women had a worse marital satisfaction than their husbands. In addition, general population studies indicate that marital and psychological distress are more common among women than men [29].

Consistent with the MDMD, the current study showed the considerable actor effect of marital satisfaction on depression, which is also consistent with previous studies conducted in USA [30], China [31, 32], Brazil [33] and Israel [12]. However, in a study conducted among Chinese older couples, neither of the actor effects was significant [34].

The key finding of our study was the link between men’s marital satisfaction and women’s depression. In line with our expectation, we found that husband’s lower marital satisfaction was associated with greater level of wives’ depression. Contrary to our expectation, the present study does not confirm a strong partner effect of women’s marital satisfaction on their husband’s depression. In other words, regarding the partner effects, the MDMD was partly supported asymmetrically among infertile couples. This type of asymmetrical pattern of partner effects has also been reported in several previous studies [34, 35]. However, contrary to our finding, Miller et al. [31] reported an opposite pattern of the partner effect in a sample of middle-aged Chinese couples.

The present research offers a number of important contributions to understanding the relationship between marital satisfaction and depression. Studies regarding this relationship tend to focus on individuals, despite the obviously dyadic nature of marital satisfaction. Many researches in this area also tend to focus on general population of adolescents and couples, most of whom have probably not yet become involved in long-term, committed marital relationships or have not yet experienced a shared health problem like infertility. The present study addresses these limitations, as it sought to test the MDMD in infertile married couples from a dyadic perspective. The findings of the present study have potentially important clinical implications. Probably one of the most key findings is the importance of taking a dyadic perspective on marital satisfaction and depression. Particularly, the study indicates that men’s marital satisfaction is important to their wives’ depression. Therapists working with infertile couples should be aware of these dyadic effects; thus, psychological interventions that target an enhancement of marital satisfaction and reduction of depression symptoms should treat the couple as a unit.

Our study had several limitations that should be considered when interpreting the findings. First, this study was conducted only in one center and therefore may not be generalizable. Second, the cross-sectional nature of the study design limits inferences about the causal relationships between marital satisfaction and depression. Longitudinal research is needed to better understand the relationship between marital satisfaction and depression symptoms, as these relationships can be multi-factorial and complex [36–38]. 

## Conclusion

Despite the limitations, the study findings provide support for the MDMD among infertile couples.

Particularly, besides the actor (intrapersonal) effects for both male and female, there were partner (interpersonal) effects of male marital satisfaction on female depression symptoms. Based on these findings, interventions to reduce depressive symptoms in infertile couples should include both husbands and wives simultaneously.

## Abbreviations

APIM: Actor–Partner Interdependence Model
EMS Scale: 10-Item ENRICH Marital Satisfaction Scale
HADS: Hospital Anxiety and Depression Scale
MDMD: Marital Discord Model of Depression
SEM: Structural Equation Modeling
